# Searching out the Secrets of Nature

**Published:** 1899-10-28

**Authors:** 


### The Hospital

A Journal of

The Medical Sciences and Hospital Administration.

Vol. XXVII.—No. 683. Edited by Sir Henry Burdett, K.C.B., and Solomon C. Smith, M.D., M.R.C.P. [October 28, 1899.

### Searching Out the Secrets of Nature.

The annual function by which the Royal College of Physicians celebrates the memory of Harvey is always an occasion of great interest, and, however lightly it may be esteemed by the juniors, is one which is treated by those who have had time and opportunity to become attached to their College in a spirit of reverence which to the onlooker is curiously suggestive of a form of worship. It is not, however, merely to pay homage to the great discoverer, nor to keep fresh the memory of their benefactor, that this large assembly of the leading physicians of the day foregathers in Pall Mall year after year on the occasion of the Harveian Oration, but rather because Harvey stands in their minds as the representative of what is the root of all medical knowledge, namely, honest, careful, and often laborious observation; and because the Oration itself is "an exhortation to the Fellows and Members of the said College to search and study out the secrets of Nature by way of experiment." We take it that, if the assembly which takes place on the occasion of the Harveian Oration has any meaning at all, it is that it stands as an annual reassertion of the faith that is in us—that medicine is not a mere empirical application of traditional remedies, that it is not a hocus-pocus allied to the witchcraft of the Middle Ages, but is the outcome of honest and scientific deduction from equally honest observation and experiment.

After describing the great advances which have been made even within very recent years by a careful persistence in these methods, Dr. Vivian Poore, the orator on this occasion, said that there have always been two schools of thought in regard to scientific inquiry. One school, fearful lest inquiry into proximate causes should lessen our reverence for the ultimate cause, protests that "all is vanity," and that "he that increaseth knowledge increaseth sorrow." The other school finds in wisdom "an unspotted mirror of the working of God." "It is," he added, "quite impossible not to admit that our increased knowledge of the laws which regulate the visible universe has increased our living faith, and added to the glory of God." Modern medicine is teaching us that much bodily suffering is due to man's wilful neglect of the beneficent laws of Nature, and the doctrine "that diseases are due to ignorance and disregard of law, and are not 'sent' as scourges by a petulant and capricious deity, is clearly a doctrine which in no way dims the glory of God." There have, however, always been trustful souls, persons with *idées fixes* to whom mental progress is painful; who see sin where none is, and who find blasphemy in the simplest acts, for, as Shakespeare has it, "there is nothing either good or bad but thinking makes it so."

"There be those," he went on to say, "who apparently hold the view that a guinea pig is of more value than many babies, and that operations on the lower animals, no matter how carefully they be done, if done for the purposes of research, diagnosis, or relief, and not with the object of making mutton tender, pigs fat, or horses quiet, are to be denounced as atrocities. With such as these it is useless to argue. But . . . . it may be well to point out that no conviction for cruelty or breach of law has ever been obtained, and that hearsay and misinterpretation of physiological writings is not evidence, at least on this side of the Channel. The money collected by these strange apostles of mercy is not spent in the law courts, where mutual cross examination would be possible, but in attempts to institute a sort of medical excommunication, and in efforts to dam the stream of charity by sophistical utterances, such as Tennyson pronounced to be 'ever the blackest of lies.'"

It is well that Dr. Vivian Poore, who, from his position as inspector under the Cruelty to Animals Act must know the actual facts of the case, should have felt himself entitled, and, what is more, should have felt himself called upon, to speak so strongly as he has done on this subject. Much mystery is often made of it; accusations fly about; assertions are boldly and unhesitatingly put forward to the effect that things are being done by physiologists which will not bear light; and the challenge now publicly thrown down by Dr. Vivian Poore is much to the point. "Bring these charges before the law courts," he practically says, "let their authors submit themselves to cross-examination, and let the truth be finally declared." This is sound advice and honest withal, and until it is taken we would insist, as Dr. Poore urges, that it behoves the medical profession to bear a wary eye lest fanatics should impose immoral tests upon applicants for hospital appointments—tests, be it remembered, which would have excluded Harvey from the service of St. Bartholomew's Hospital. "No hospital governor," he adds, "who coquets with tests of this kind can possibly command the respect of our College, nor can we admit that such an attitude towards research can do anything but harm to the cause of the suffering poor."

